# Deletion Mutants, Archived Transposon Library, and Tagged Protein Constructs of the Model Sulfate-Reducing Bacterium Desulfovibrio vulgaris Hildenborough

**DOI:** 10.1128/MRA.00072-21

**Published:** 2021-03-18

**Authors:** Judy D. Wall, Grant M. Zane, Thomas R. Juba, Jennifer V. Kuehl, Jayashree Ray, Swapnil R. Chhabra, Valentine V. Trotter, Maxim Shatsky, Kara B. De León, Kimberly L. Keller, Kelly S. Bender, Gareth Butland, Adam P. Arkin, Adam M. Deutschbauer

**Affiliations:** a Biochemistry Division, University of Missouri, Columbia, Missouri, USA; b Physical Biosciences Division, Lawrence Berkeley National Laboratory, Berkeley, California, USA; c Environmental Genomics and Systems Biology Division, Lawrence Berkeley National Laboratory, Berkeley, California, USA; d Life Sciences Division, Lawrence Berkeley National Laboratory, Berkeley, California, USA; Indiana University, Bloomington

## Abstract

The dissimilatory sulfate-reducing deltaproteobacterium Desulfovibrio vulgaris Hildenborough (ATCC 29579) was chosen by the research collaboration ENIGMA to explore tools and protocols for bringing this anaerobe to model status. Here, we describe a collection of genetic constructs generated by ENIGMA that are available to the research community.

## ANNOUNCEMENT

The study of sulfate-reducing microorganisms (SRMs) is necessitated by the multitude of impacts caused by their metabolism on Earth’s sulfur, oxygen, and carbon cycles (1, 2), their corrosion of concrete and ferrous metal structures (3, 4), and their involvement in human health (5). Recently, possible SRM contributions to biohydrogen and hydrocarbons for biofuel, polyhydroxyalkanoates to replace plastics, bioremediation of toxic metals, and bioactive metal sulfides for cancer treatment have been revealed (2). With 28 mM sulfate in the oceans, SRMs have a competitive advantage for biomass turnover there. Estimates of SRM degradation of about 50% of the organic matter reaching the marine sediment (1, 6) would make them among the more abundant organisms on Earth.

Because of the environmental importance of these microbes, the first sulfate-reducing bacterium with a sequenced genome, Desulfovibrio vulgaris Hildenborough (7), was chosen to be brought to model status for use in generating a transposon (Tn) library and for constructing strains that produce affinity-tagged proteins (Table 1). In the Dryad digital repository, we provide a list of the Hildenborough constructs and a ReadMe file that describes their construction (9). Below, references are included where construction details can be found.

**TABLE 1:** Summary of genetic constructs of Desulfovibrio vulgaris Hildenborough

Type of construct	Total no. of constructs	No. of unique genes mutated	% of predicted protein-encoding genes
Marked and unmarked gene deletions^*a*^	>725		
Tn mutants	11,034	2,480	72.6^*b*^
Barcoded deletions for expanded Tn mutant pool	275	214	6.3


	Total no. of constructs	No. of tags	% of predicted protein-encoding genes modified
Constructs for tagged proteins	1,568^*c*^		45.9
STF		1,231	36.0
STF-6× His		237	6.9
SNAP		61	1.8
Other tags		39	1.1

a Includes marker-replacement deletions (MR), marker-less deletions (MLD), site-directed mutants, and a few complementation strains.

b Total of 3,417 (8).

c Total of 1,430 unique genes.

Marker exchange mutation, replacing a nucleotide sequence with a selectable marker flanked by homologous chromosomal regions, has been the cornerstone of genetic constructions (10, 11). To generate in-frame deletions without a residual selectable marker, a parental strain, JW710, was created that is resistant to inhibition by 5-fluorouracil through deletion of the uracil phosphoribosyltransferase (*upp*) gene (12). The return of the *upp* gene restores sensitivity, providing a counterselectable gene. JW710 also allows site-directed mutations (13) and multiple deletions to be created without an accumulation of selectable markers (12, 14). The plasmids, pSC27 (15), pMO719 (16), pMO9075 (11), and pMO746 (17), with features used in various strain constructions, are available from https://www.addgene.org/Judy_Wall/.

Transposon mutants were generated by conjugation of a nonreplicating plasmid encoding a mini-Tn*5* (18) conferring kanamycin (and Geneticin) resistance and modified with barcoding oligonucleotide sequences (19, 20). Kanamycin-resistant transconjugants were recovered from transposition events and subjected to sequencing to locate the insertion site of the transposed DNA. Over 10,000 transconjugants were isolated and archived individually. Pools of these transposon mutants marked with TagModules were made to assay gene fitness in parallel (19), but their use revealed that unidentified members had an aerobic contaminant(s). Confirmation of the axenic status and the genome location of the transposon should be performed prior to use of these mutants. Randomly barcoded TnSeq approaches (21) have since been used to make pooled transposon libraries successfully in Hildenborough. These pooled bar-coded transposon mutants are also available upon request (22; V. Trotter, personal communication, 27 August 2020).

Affinity-tagged gene constructs were made for affinity purification to identify protein-protein interactions (23, 24). Sequential tags were Strep-tag (25) and FLAG (26), separated by the tobacco etch virus (TEV) protease site (27), referred to as STF or STF with 6× His for C-terminal tagging. Single-tagged constructs with SNAP allowed *in vivo* covalent tagging with a fluorescent dye (28).

### Data availability.

The genetic constructs described herein are available, within reason, from Valentine V. Trotter (vvtrotter@lbl.gov) and Adam M. Deutschbauer (amdeutschbauer@lbl.gov). The constructs can be found listed at the Dryad digital repository (https://doi.org/10.5061/dryad.h70rxwdh9). These strains were generated, wholly or in part, in the laboratory of Judy D. Wall, and the list was deposited in 2021. A ZIP file was deposited at Dryad containing a ReadMe document, a composite Excel file, an Excel file necessary for TagModule/barcode identification of transposon mutants and of complementary barcoded gene deletions, and a text file listing of TagModules. On sheet 1 in the composite Excel file is a search engine that will identify all constructs available for any gene locus provided as a DVU number.
